# Trust, conversations and the ‘middle space’: A qualitative exploration of the experiences of physiotherapists with clients with suicidal thoughts and behaviours

**DOI:** 10.1371/journal.pone.0238884

**Published:** 2020-09-10

**Authors:** Ryan L. McGrath, Tracey Parnell, Sarah Verdon, Jasmine B. MacDonald, Megan Smith

**Affiliations:** 1 School of Community Health, Charles Sturt University, Albury, NSW, Australia; 2 Discipline of Psychology, RMIT University, Melbourne, VIC, Australia; 3 School of Psychology, Charles Sturt University, Wagga Wagga, NSW, Australia; 4 Faculty of Science, Charles Sturt University, Wagga Wagga, NSW, Australia; Newcastle University, AUSTRALIA

## Abstract

In Australia, physiotherapists are registered healthcare practitioners who possess the knowledge and skills to care for clients with poor physical health as a result of musculoskeletal, neurological, and respiratory conditions. Although physiotherapists are not considered a primary profession in the Australian mental health workforce, the association between suicide and poor physical health suggests that they may encounter clients with suicidal thoughts and behaviours. We used a qualitative approach inspired by phenomenology to explore the experiences of nine physiotherapists who encountered clients with suicidal thoughts and behaviours. We used a combination of focus groups and in-depth interviews to collect this data. The data were analysed inductively using framework analysis. The main themes identified in the data were: i) the importance of trust, ii) the mechanism of conversation, and iii) the 'middle space'. The middle space refers to the experience of working with clients at risk of low or medium risk of suicide. A trusting practitioner-client relationship was reported to be essential in facilitating the disclosure of suicidal thoughts and behaviours. Physiotherapists also reported that less structured subjective assessments encourage clients to talk more openly, which in turn facilitates the disclosure of suicidal thoughts and behaviours. Once the disclosure of suicidal thoughts and behaviours occurred, physiotherapists reported a lack of confidence regarding role clarity and issues associated with this. Difficulties were most evident during encounters with clients with low to medium suicide risk due to a lack of confidence in the accuracy of assessment of these clients. The findings suggest that physiotherapists are well placed to detect and/or receive disclosure of suicidal thoughts and behaviours, as well as the need for physiotherapists to be trained in how to support clients who disclose suicidal thoughts and behaviours.

## Introduction

The high incidence of suicide is of concern to health departments, advisory bodies and society around the world [1]. In Australia, the National Suicide Prevention Adviser [2] highlighted the need for better coordination of health responses to suicidal thoughts and behaviours. Although death by suicide is a commonly used metric to measure the impact of suicidal thoughts and behaviours, the number of completed suicides in Australia in 2018 (3,046) pale in comparison to the estimated number of incomplete suicides (65,000), suicide plans (91,000) and suicidal ideation (370,000) that were recorded [3].

Existing suicide prevention policies, such as the *Framework for Suicide Risk Assessment and Management for New South Wales Health Staff*, expect that all health staff should be able to conduct a preliminary suicide risk assessment [4]. Policies in other Australian states, such as the *Victorian Suicide Prevention Framework 2016–25*, advocate for the training of people who are likely to encounter at-risk individuals [5]. Both the New South Wales (NSW) and Victorian suicide prevention frameworks focus on reducing the number of deaths by suicide [4, 5], yet many more people are affected by suicidal thoughts and behaviours.

It has been argued that suicide prevention strategies should move away from focusing on those at high risk of suicide and instead aim to identify people at all stages of the suicide trajectory [6–8]. Although risk factors do not necessarily predict whether an individual will complete suicide, they are useful in identifying groups at risk of suicide [9, 10]. Physical health problems have been identified as a risk factor for suicide [11]. A study completed in 2006 by the Commonwealth of Australia (as cited in [12]) found that physical health problems were the most influential life event in 21% of completed suicides. The association between suicide and poor physical health suggests that a broad range of healthcare practitioners, not just mental health professionals, may encounter clients with suicidal thoughts and behaviours. For this study, we used the term suicidal thoughts and behaviours to refer to the spectrum of suicidality which includes complete suicide, incomplete suicide, suicide plans, suicidal ideation, intentional self-harm and death wishes [13, 14].

In Australia, physiotherapists are registered, first-contact health practitioners who typically treat clients with injuries, pain, diseases, disorders and conditions that affect their function and movement [15]. Physiotherapy is the largest health profession not considered a primary profession in the Australian mental health workforce [16, 17]. However, physiotherapists are explicitly mentioned by NSW Health [4, p. 4] as one group of health practitioners that "must be able to perform a preliminary suicide risk assessment." As part of a preliminary suicide risk assessment, physiotherapists are expected to conduct a "brief psychiatric assessment" and make an "assessment of suicide risk" of any clients they suspect of being at risk of suicide [4, p. 4]. A brief psychiatric assessment involves the identification of psychiatric symptoms such as the symptoms of depression and an assessment of coping skills [4]. An assessment of suicide risk involves asking screening questions such as "have things been so bad lately that you have thought you would rather not be here?" [4, p. 4].

Although not considered a primary mental health profession, the role of physiotherapy in mental health has received increased attention in the past decade [18–21]. Mental health physiotherapy is a specialty within the profession that involves physiotherapists addressing the physical health needs of people with mental health conditions [19, 22]. Much of the research in the area of mental health physiotherapy has focused on clients with diagnosed mental health conditions [21, 23, 24]. According to the fifth edition of the Diagnostic and Statistical Manual of Mental Disorders, suicidal behaviour is not a diagnosis [25]. However, it has been argued that suicidal behaviour should be considered a distinct diagnosis, as suicide may occur in the absence of other mental health conditions [26–28]. For this study, we considered suicidal thoughts and suicidal behaviours as health issues in their own right.

We completed a search of the existing literature and noted a paucity of research related to physiotherapy and clients with suicidal thoughts and behaviours. However, McVey et al. [29] completed two multi-professional tweetchats that involved physiotherapists, other healthcare practitioners and the general public. McVey et al. [29, p. eS132] found that "some" of the participating physiotherapists reported a lack of confidence in suicide risk assessment. The literature search also identified four case reports [30–33] and a qualitative study [34] that mentioned physiotherapy involvement with clients with suicidal thoughts and behaviours; however, suicidal thoughts and behaviours were not the primary focus of these studies. We were unable to locate any peer-reviewed studies that specifically explored the experiences of physiotherapists who encounter clients with suicidal thoughts and behaviours. In this study, we aimed to understand how physiotherapists recalled, reflected and perceived their experiences of working with clients with suicidal thoughts and behaviours. Our research question was:

What are the experiences of physiotherapists who encounter clients that they perceived as having suicidal thoughts and behaviours?

For conciseness, we referred to the phenomenon of interest as 'the experiences of physiotherapists with clients with suicidal thoughts and behaviours' hereafter.

## Method

This study was reported in accordance with the Standards for Reporting Qualitative Research [35] (S1 Table) and was approved by the Human Research Ethics Committee of Charles Sturt University (Protocol number H18234). Written informed consent was obtained from all participants. No participants reported distress arising from the research.

### Methodological framework

This qualitative study was conceptualised within the ontology of social constructivism. This meant we aimed to understand the human experience of the situation being studied by exploring the perception of the participants [36] while recognising that all experiences are subjective [37]. At an epistemological level, we drew on the work of phenomenologists van Manen [38] and Spiegelberg [39]. At its heart, van Manen argues that phenomenology is concerned with "the lived world as experienced in everyday situations and relations" and the purpose of the phenomenological analysis is to uncover the meaning of the experience [38, p.101, 40]. We felt that van Manen's perspective regarding the lifeworld and his approach towards analysis was well suited to our research question. We also drew on the work of Spiegelberg [39] to help guide us through the practice of cooperative phenomenology. Traditionally, phenomenological studies have used individual interviews to ensure that the participant's experience is captured and remains uncontaminated by the experiences of other participants; as would occur in a focus group [41]. Conversely, from a cooperative phenomenological perspective, this 'contamination' of multiple participants' experiences is not seen as incongruent with phenomenology [42]. Instead, the emphasis on collaboration and dialogue that is inherent to cooperative phenomenology is thought to open up new perspectives and encourage discussion that may not have emerged from individual interviews [39]. The dynamic process of group discussion also stimulates the openness of the researcher, aiding them to identify a broad range of perspectives about the subject [39]. Because of the inherent benefits of cooperative phenomenology regarding rigour and trustworthiness, we felt that its inclusion was justified. The social constructivist’s view that an individual's reality is subjective and the cooperative phenomenologist’s perspective that group phenomenology 'intersubjectivises' subjective knowledge is not only congruent but complementary [37, 39, 42–45].

Although we drew on the work of van Manen [38] and Spiegelberg [39], we departed from a strict adherence to phenomenology as the data revealed important information about the actions of physiotherapists and what led them to behave in a particular way. The inclusion of the action-orientated data as a part of the first and second theme was essential in understanding and conceptualising the third theme, yet the first and second theme may not be considered true phenomenological findings. As van Manen puts it, "not all qualitative research inspired by phenomenology is phenomenology" [46, p. 777]. By analysing this data, we were able to not only understand the experience as it is lived but also understand how the experience was interpreted.

### Participants, recruitment and sampling

**Fig 1:**
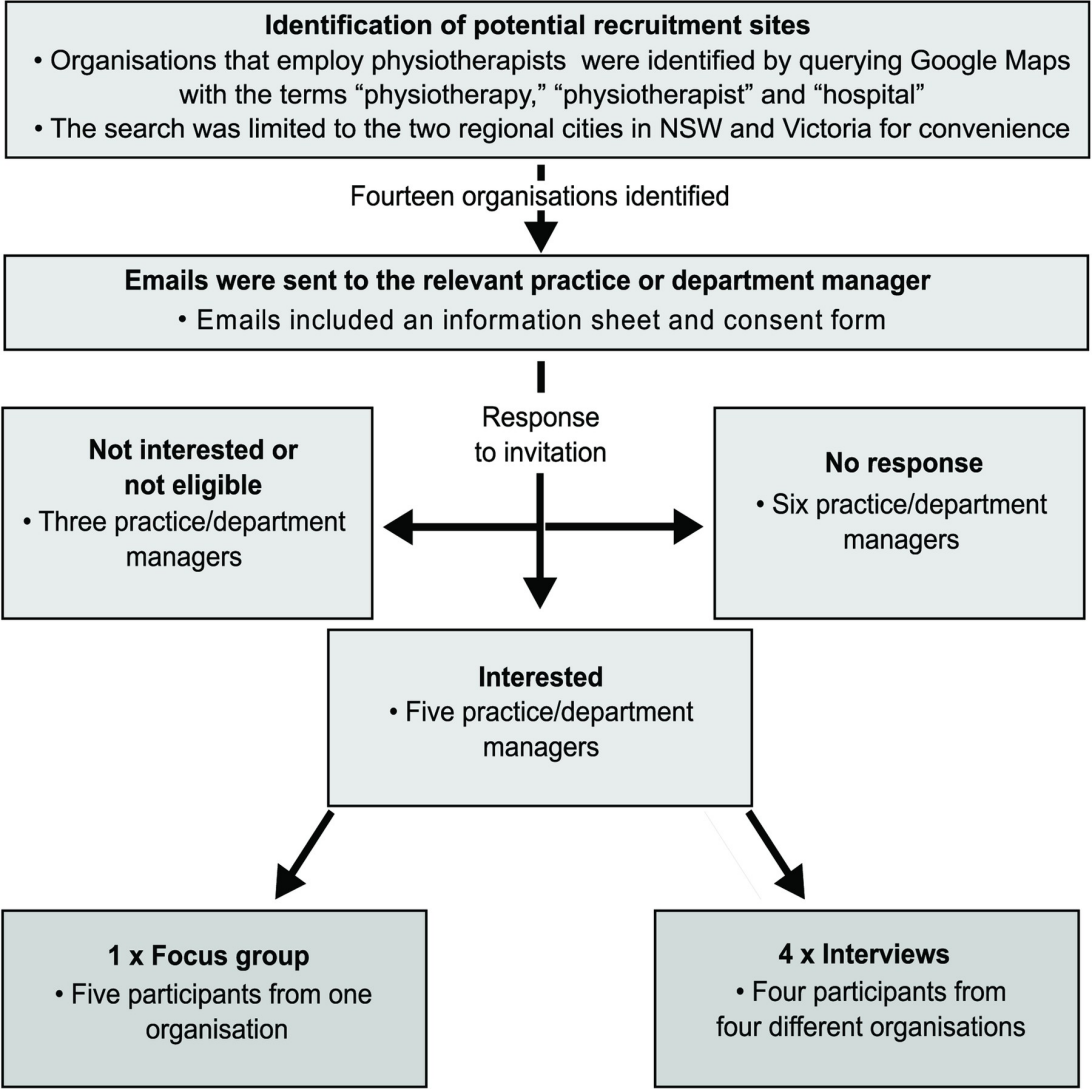
The recruitment process for the focus group and interviews.

Nine registered and practising physiotherapists (6 females, 3 males; mean age = 38 years, SD = 10) from two cities in regional NSW and Victoria were recruited using purposive and convenience sampling (Fig 1). To participate, physiotherapists had to be currently working as a clinical physiotherapist, and self-report having treated clients they perceived as having suicidal thoughts and behaviours. Five participants were employed at one practice (pseudonym: Clinic Physio), and the remaining participants were from four different private practices. Four of the five private practices (including Clinic Physio) offered general physiotherapy services. The other private practice specialised in disability and aged care. The education levels of the participants ranged from bachelor to doctoral level (mean Australian Qualifications Framework Level = 8, SD = 1.0), and mean years of clinical experience was 12 years (SD = 8.8). Due to participant response and availability, one focus group and four individual interviews were conducted. The focus group consisted of all five participants employed at Clinic Physio. The small recruitment pool of physiotherapists and the unique demographic information of some of the participants resulted in the decision not to publish individual participant demographic data.

Recruitment was ceased on pragmatic grounds (resource and time constraints). No new themes emerged in the final two interviews, suggesting that data saturation may have been reached. Although small nuances within each theme were noted during data analysis, the number of participants created sufficient data to address the aim of the study.

### Data collection

The first author conducted the focus group and interviews. The focus group and interviews were audio-recorded, and ran for approximately two and one hours, respectively. The focus group followed the rules of group discussion outlined by Spiegelberg [39] to ensure each participant's experience was captured.

### Data analysis

**Fig 2:**
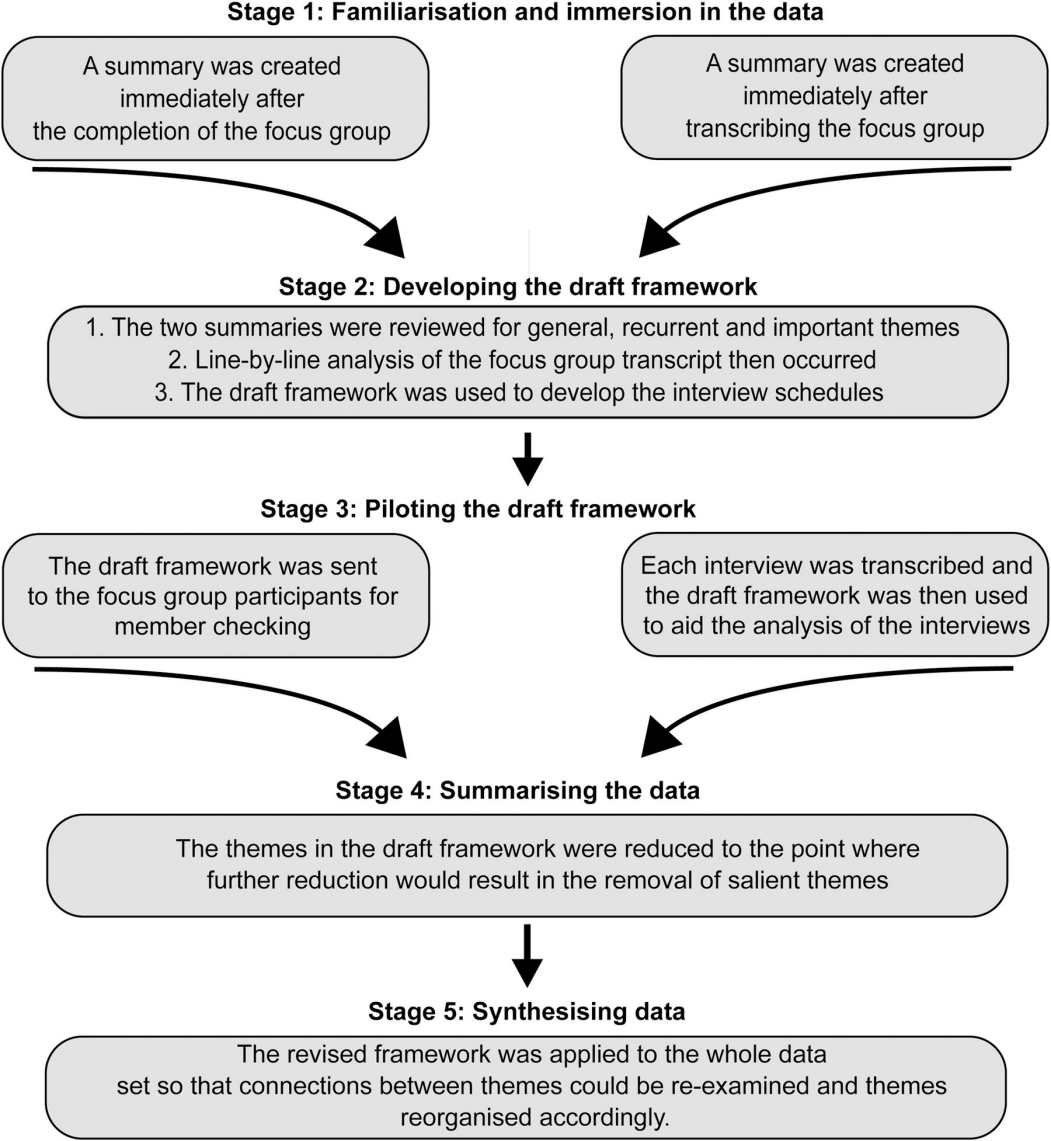
The tailored version of the framework analysis used.

The audio recordings of the focus group and interviews were transcribed by a third-party transcription service and reviewed by the first author for accuracy. Data analysis followed the principles of van Manen's [38] phenomenological philosophy, starting with engaging with the data in its entirety so that a central meaning of the experience can be distilled. A line-by-line method of analysis, as described by van Manen [38] was then completed. The line-by-line method involved each sentence being examined for information about the phenomenon. An adapted version of framework analysis (Fig 2) as described by Ward et al. [47] was used to bring together van Manen's [46] phenomenology and cooperative phenomenology [39] to ensure that the analysis was iterative, rigorous and transparent. The focus group was analysed before the interviews were conducted in order to create a draft framework. The draft framework served as the point of reference for all subsequent interviews. NVivo version 10 and Word 365 was used to manage the data.

### Rigour/trustworthiness

The use of focus groups and interviews in this study allowed us to triangulate the data and increase the trustworthiness of the findings. The trustworthiness of the findings was further improved through member checking. We emailed participants the summary of the focus group and asked for feedback regarding our interpretation. Their feedback was used to further refine our analysis. Interview participants were not given the opportunity to provide feedback because the findings from each interview were synthesised with findings from the focus group during analysis.

The first author kept a reflexive journal which was re-examined throughout the analysis phase to limit the effect of researcher partiality. However, no matter how much the researcher self-scrutinises, the experiences of the participants will always be presented through the "reflective and discursive lens of the researcher" [48, p. 973]. The research team also consisted of researchers from diverse training and practice backgrounds, including physiotherapy, occupational therapy, psychology, social work, sociology, and speech therapy. These differences in the individual researchers' lens further strengthened the rigour of the analysis.

## Findings and discussion

**Fig 3:**
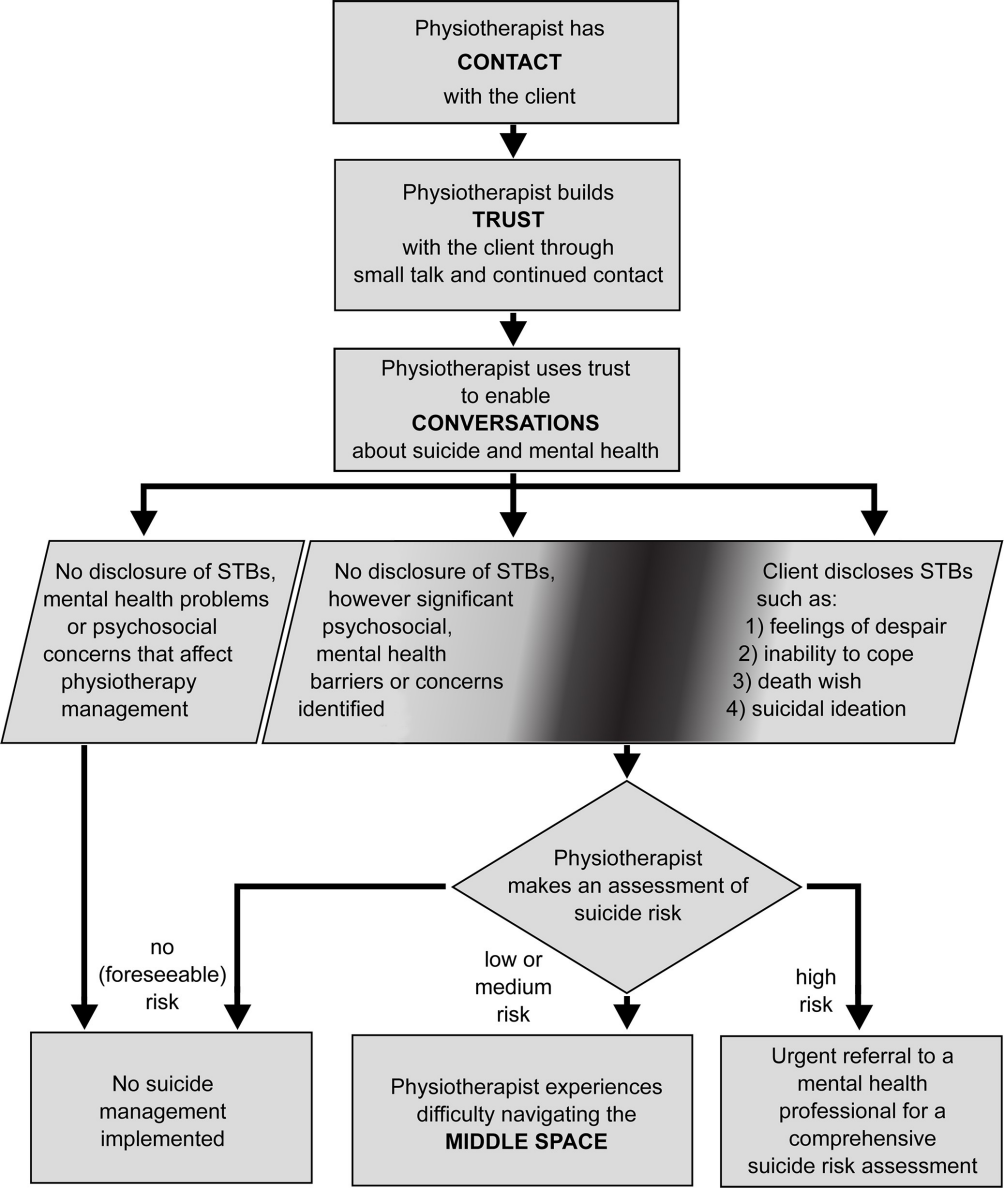
Overview of the relationship between the key themes.

The findings revealed that the participants were sensitive to the psychological distress of their clients and made a deliberate effort to talk with clients about how they were coping with their physical conditions. The three main themes identified were: i) the importance of trust, ii) the mechanism of conversation, and iii) the 'middle space'. Fig 3 provides an overview of the themes and demonstrates that when the data are viewed as a whole that the themes told a story. Although linking the themes together as a flowchart may suggest a linear process, the findings also showed that participants could move in and out of different stages and could be within two or more stages at once.

### Theme 1: The importance of trust

*It's all about establishing that trust*. *(Focus Group Participant [FGP]-1)*

The first theme that emerged from the participants' experiences of working with clients with suicidal thoughts and behaviours was the importance of trust in the practitioner-client relationship. Participants reported that building a trusting relationship with their client was an essential part of being an effective physiotherapist and was something they deliberately tried to develop. Through developing and maintaining trust, participants found themselves in the position to detect or receive disclosure of suicidal thoughts and behaviours.

*People require trust to be able to openly talk about [their mental health]*. *If I feel like there is something going on there*, *then I want them to feel comfortable to open up and not feel like they're getting pried at or interrogated*. *(Interview Participant [IP]-1)*

Without this trust, participants speculated that clients would not self-disclose suicidal thoughts and behaviours to the physiotherapist.

*If people don't have trust in their health professionals*, *I think that's when people slip through the cracks and don't ask for help*. *(FGP-2)*

Multiple participants used the word "trust" and the phrase "open up" in close succession. This highlighted the importance of trust in facilitating disclosure of issues impacting them, including suicidal thoughts and behaviours.

*It's all about establishing that trust in that relationship*, *which is probably why people will open up to you about how they're feeling; they may not have disclosed [suicidal thoughts and behaviours] to other people*. *(FGP-1)*

Small talk was the primary process through which trust was developed. Small talk refers to conversations that are considered off-task and involve suspension from institutional roles (that is, the roles of client and physiotherapist) [49]. Participants felt that, because small talk was more personal than clinical, they were better able to build rapport with their clients.

*A lot of what we do is just general chit-chat dialogue and getting to know people… While we are doing the manual [therapy] … we enjoy knowing [who] we are treating and getting to know them and finding out about their family … They often find out about ours*, *too*, *I suppose*. *You might see them every week for three to six or eight weeks*, *so they have that ability to form that relationship and that trust with you*. *(FGP-3)*

In addition to the role of small talk in the development of trust, the above quote also highlighted how multiple consultations could facilitate the development of trust. Participants reported waiting for multiple consultations to pass before asking about their client's mental health and suicidal thoughts and behaviours. This deliberate delay in questioning was due to the participants' fear of compromising the relationship.

*If it's past the third or fourth session that I'm seeing them*, *then I feel like I've developed a close enough rapport to be able to say*, *"You're looking a bit flat today, or looking a little bit down*.*" … I want to feel confident that I have a bit of their trust… first*. *(IP-1)*

One participant described the development of trust as being important to avoid offending or making clients feel uncomfortable about disclosing mental health problems and suicidal thoughts and behaviours.

*You can get people offside very quickly and make them feel a lot worse emotionally [when talking about mental health and suicidal thoughts and behaviours]…*. *I think that unless they're showing lots of flags that they're really planning something imminently*, *I would be wanting to probably see them a couple of times before I'd be saying*, *"Look*, *I think that this mental health stuff is getting in the way*, *maybe more than the physical stuff*, *and we probably need to make a bit of a plan and talk about that"… You've got to have that trust and…connection with them*. *(IP-2)*

However, another participant shared an experience where little to no trust had been developed, but where it was necessary to address suicidal thoughts and behaviours as a matter of urgency. This participant's experience involved a client who presented for an initial consultation with drug intoxication and obvious suicidal thoughts and behaviours.

*[The client] was completely off his head and on a whole lot of medications*. *He was quite open about it*: *He was taking his friend's Lyrica*. *He had back pain*, *maybe*. *I think when he walked out of the room*, *he threw up*, *but he was actually in and out of consciousness during that appointment*. *We ended up calling Mental Health [Access Line]*, *and I said to him*, *"Are you having thoughts [of suicide]?" He said "Yes*.*" (FGP-4)*

The above experiences highlight the importance physiotherapists place in developing and maintaining a trusting practitioner-client relationship. When considering the theme in the context of a suicide risk assessment, an effective assessment appears to be more than just following the protocol for mandatory assessment. Other researchers have identified the importance of trust as a key facilitator for the disclosure of suicidal thoughts and behaviours. Ganzini et al. [50] found that veterans were more likely to disclose suicidal thoughts and behaviours to healthcare practitioners who they trusted and knew well. Ganzini et al. [50] also found that a lack of a continuous relationship with a healthcare practitioner was a barrier to the disclosure of suicidal thoughts and behaviours. Based on the experiences of the participants in this study and Ganzini et al. [50], it appears that in most cases, trust is essential for the disclosure to occur.

Participants used small talk to establish trust and develop a connection with clients. An analysis of 39 consultations between medical practitioners and clients with breast cancer found that clients valued having a relationship with their medical practitioner [51]. The study also revealed that a brief conversation unrelated to the client's health condition was one strategy used to develop this relationship [51]. Wright et al. [51] argued that based on their findings, a model of clinical communication that emphasises the need for a trusting, caring relationship between practitioners and clients is needed. Building trusting practitioner-patient relationships is a key feature of the relationship-centred care model [52]. Relationship-centred care is not a new concept to physiotherapy practice [53]. An analysis of 52 consultations found that the physiotherapist-client interaction more closely resembled relationship-centred care than either patient-centred care or the biopsychosocial approach [53]. Similarly, an investigation of the perspectives of physiotherapists after cognitive functional therapy training found that these physiotherapists believed that trust would also help their clients open up and facilitate the disclosure of sensitive information [54]. Based on the findings of our study, as well as previous research, it appears that physiotherapists are already making the development of trust a priority in their consultations. Because of this trust, clients are likely to disclose sensitive information, including suicidal thoughts and behaviours, to their physiotherapist.

### Theme 2: The mechanism of conversation

*Through the mechanism of conversation*, *we tend to find out [about their suicidal thoughts and behaviours]*. *(FGP-3)*

Although most participants reported that they were not actively screening for suicide, they reported intentionally modifying their consultations to be more conversational in order to create an environment where clients could more easily disclose problems.

*[Suicide] is not something I actively screen for; it's something that I hope emerges in an open-ended discussion*. *(IP-3)*

Client-led conversation was one strategy adopted by participants. Client-led conversations referred to conversations where the clients chose the topic of conversation. Participants felt that client-led conversations enabled clients to talk about what was important to them at that point in time. Participants reported that sometimes the client's pressing concern happened to be their suicidal thoughts and behaviours. However, even though participants hoped that clients would disclose their suicidal thoughts and behaviours if they had them, participants still found a client's disclosure of suicidal thoughts and behaviours to be confronting.

*When somebody is so forthright*, *[in] saying that [they] wanna die or [they] wanna kill [themself] … you panic*. *(IP-2)*

Participants reported that early in their careers, the highly structured clinical assessment they were taught hindered their ability to complete a holistic assessment and thus needed modification. Participants suggested that the transition from a more structured interview style to a more conversational interview style was made easier once they became more proficient at remembering the subjective questions that form the basis of a physiotherapy assessment.

*The more experienced the physio gets in general conversation… they become able to pick up so much more about somebody's mental health as well as their physical health*. *(FGP-2)*

For example, IP-3 had chosen to move away from using forms and completing clinical notes during consultations; opting to incorporate relevant clinical questions into general conversation and writing the clinical notes after the consultation.

*From my experience*, *we teach grads too much how to be too recipe-based… Ask about pain*, *ask about function*, *ask about this*. *It's very much sort of tick boxes*. *That's not how people operate*. *That's not how conversations operate… What I've learned more about performing a good interview and a good subjective assessment [is that] if you can be more open and more natural then it feels more like a trusting two-way relationship…*. *I tend not to write any notes during my session anymore*. *I write all my notes after I've completed the session*. *(IP-3)*

Participants also described practitioner-led conversations that could result in clients disclosing suicidal thoughts and behaviours. Participants felt it was important to initiate a discussion with clients about how they were coping with their injury and ask about any barriers impeding their recovery.

*We're trying to identify barriers…*. *[It] might just happen to be that [the client's mental state is] also threatening their life*. *I wouldn't say that it is an incidental [finding]*, *but it's not necessarily the answer you're after*. *(FGP-3)*

Although not specific to suicide, other authors have highlighted the importance of less structured conversations in healthcare and specifically physiotherapy. MacDonald [55] examined the clinical encounters of two nurses over approximately two-and-a-half hours and found that small talk was able to be used effectively to gather clinically useful information. For example, one nurse used small talk to explore how the client was coping with their medical condition [55], which was similar to how participants in the current study used practitioner-led small talk. Another study by Angus et al. [56] found that clinical conversations between physicians and clients that involved small talk and fewer closed questions uncovered more information about a client's wellbeing than through rigid checklist-style consultations.

In the context of physiotherapy, Cowell et al. [54] found that after cognitive functional training, physiotherapists moved away from a structured approach to a quasi‐conversational style because they perceived that it would help clients voice their concerns. These findings are similar to the experiences of the participants in the current study, who have intentionally moved away from rigid formalised consultations to more informal, conversational-style consultations. Benwell et al. [49] discussed how small talk could evolve into a client disclosing personal problems; however, they also found that this type of small talk can disrupt the completion of essential tasks. The experiences of the participants in this study were that open, free-flowing client- or practitioner-led conversations, in the context of a physiotherapy consultation, may facilitate the disclosure of suicidal thoughts and behaviours. Consequently, these styles of communication in physiotherapy may be a unique opportunity for suicide prevention. However, a client's disclosure of suicidal thoughts and behaviours can be unexpected and confronting for physiotherapists; this is further explored in the following theme.

### Theme 3: The middle space

*It's hard to delineate where… that boundary lies*. *(FGP-1)*

Participants reported encountering clients across the entire spectrum of suicidal thoughts and behaviours. All participants had encountered a client with a mental state that was a risk factor for suicide (for example, feelings of despair and hopelessness). Participants had also encountered clients who made seemingly flippant comments about wanting to die, clients with thoughts of suicide, and clients who had disclosed plans for suicide. The spectrum of suicidal thoughts and behaviours and the relationship between mental health problems and suicide was challenging for participants.

Participants felt equipped to assess and manage clients that they perceived as being at high or immediate risk of suicide. Participants recognised that these clients required immediate referral to a mental health professional to ensure the client’s safety.

*Have they had thoughts of harming themselves or ending their life? Personally*, *I'm reasonably comfortable asking those questions…*. *If they have divulged that information… [I then create] a plan with their [general practitioner] or the mental health team… so that they have that next course of action in place*. *(FGP-1)*

**Fig 4:**
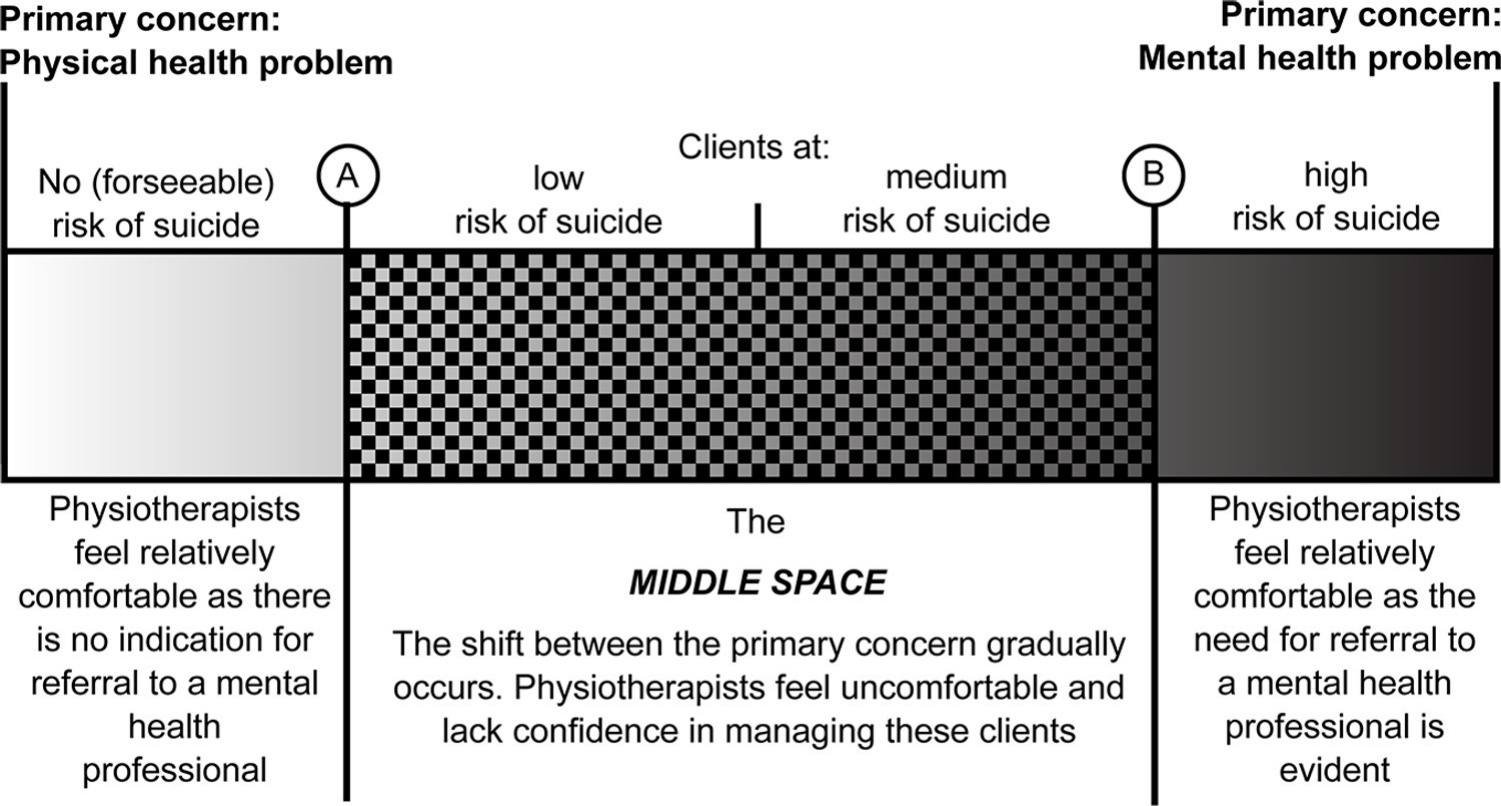
The middle space. Although the severity of mental health problems and suicide risk are correlated [58], this figure does not suggest that a client with a severe mental health problem must be at a high risk of suicide or vice versa.

Participants also felt confident in managing clients with no identified risk of suicide as managing these clients did not require knowledge of mental health or suicide risk assessment. However, there seemed to be a threshold where participants became less confident in their ability to manage the client (point A on Fig 4). A second threshold also existed where confidence in their ability to manage the client returned (point B on Fig 4). The area between these two thresholds will hereafter be referred to as the 'middle space'. The term, ‘middle space’ originated with the participants as a way to describe an area in the centre of a broader spectrum that is defined by the relative dominance of physical and mental health conditions and the presence of suicidal thoughts and behaviours; as perceived by the participant. However, a search of the grey literature revealed that the term the middle space has also been used in the context of physiotherapy to describe treatments that fall between traditional physical therapies and psychological therapies [57]. Although similar, the middle space in our study was not defined by treatment.

An example of a participant having difficulty assessing a client in the middle space was FGP-3. FGP-3 recounted an experience in which they were unsure if a client’s comment was a flippant comment or a genuine disclosure of a suicidal thought.

*[A client] made a comment that [his] farm was not doing well…*. *[He] then said something about*, *"Oh*, *I just need a bullet to the head*.*" I thought about that once I left work…*, *"Was that real? Is that really how you feel?" It's hard to know sometimes*. *(FGP-4)*

Similarly, FGP-2 reported difficulty assessing client’s in the middle space. FGP-2’s partner worked in the emergency services. Consequently, FGP-2 would sometimes hear about clients who had completed suicide. These clients would often have chronic pain and be taking prescription medication; both are identified risk factors for suicide [4, 59]. Chronic pain is also associated with depression which is also a risk factor for suicide [4, 60]. FGP-2 felt that these clients were difficult to assess because they had not explicitly disclosed suicidal thoughts and behaviours.

*They are often the ones I haven’t picked… [Hearing about their suicide is] a shock to me… I think we are able to help those who do verbalise their [suicidal thoughts and behaviours]*. *(FGP-2)*

The lack of confidence in assessing and managing clients with suspected or confirmed suicidal thoughts and behaviours resulted in notable discomfort for the participants. The discomfort ranged from slight momentary uneasiness to prolonged emotional distress.

*I'll wake up in the middle of the night worrying*, *thinking*, *"Shit*, *I should have asked that question*, *or I should have followed up with this*.*" (IP-3)*

This discomfort reported by participants was largely attributed to a lack of role clarity and formal training in suicide risk assessment.

*It's very hard to get confidence in the fact that it is actually your job*, *and you do actually have a role to play in assessing people's mental health*. *Second of all*, *you don't have any confidence in what to actually do and how to actually do it… As for my education*, *there was zero mention of [suicidal thoughts and behaviours]*. *The closest to that sort of thing when I was at uni was you should do your Mental Health First Aid*. *(IP-2)*

*I feel… uncomfortable that I don't… have the tools to ask [those questions] and to assess [the clients] risk [of suicide] … I feel silly [saying] … "Oh yeah*, *thanks for telling me*. *And tell me more about your leg*.*" I feel like it's really dismissive because I don't know where to go*. *(IP-3)*

Multiple participants reported that they ended up acting as more of a counsellor than a physiotherapist with clients who they perceived were at low or medium risk of suicide.

*We sometimes do get caught up in becoming counsellors*, *and we're not equipped to be counsellors or mental health workers*. *We're good at*, *I think*, *screening and being able to see if somebody's at risk [of suicide]…*. *Hopefully [we are also good at referring] them [to a mental health professional]*, *but knowing our limitations is so important*. *(FGP-2)*

*How do you draw that line?* *(Facilitator)*

*I find that really difficult*. *(FGP-1)*

One explanation offered by participants as to why they would become involved in discussions about mental health was because their practice was influenced by the biopsychosocial model, and therefore, they were interested in how their client was coping with their physical condition.

*[I] establish in their initial consult how they will cope with [their injury] emotionally and psychologically*. *(FGP-5)*

Another explanation was that participants felt that some clients would discuss mental health problems with them because these clients viewed physiotherapists as offering more than physical therapy and support.

*People see us as pseudo-psychologists and counsellors because we do a little bit of side training*, *but we're certainly not trained counsellors*. *My biggest fear… is that people see me as that too much*. *(FGP-2)*

A common topic of conversation between participants and their clients related to the clients' feelings of despair. One participant would commonly find themselves engaging in conversations about loss and despair.

*[My clients in aged care] experience a loss of identity [and] a loss of independence*. *All those things come up quite often*. *There are people [in aged care] that really struggle with that… There has been a person*, *in the last two weeks*, *in one of our aged care facilities trying to commit suicide…*. *[Suicidal thoughts and behaviours are] something we are dealing with on a day-to-day basis*. *(IP-4)*

Participants reported using psychological interventions when clients had psychosocial problems related to their physical health condition. However, participants did not offer psychological interventions to clients at high risk of suicide or were experiencing severe mental illness. Psychological interventions in the context of physiotherapy practice are referred to broadly as psychologically informed practice [61].

*I talk to patients about their anxiety and depression which obviously ties into chronic pain and giving them strategies to work with whether that'd be breathing or whether that'd be talking about some of the apps that are available that they can utilise…*. *Not as a standalone; we're not trying to be a psychologist*, *but that's I suppose where the lines blur a little bit [in that] we're giving people those sorts of strategies*. *(FGP-1)*

Participants reported feeling more confident navigating the middle space as they became more experienced clinicians. However, this increased confidence was primarily attributed to improved interpersonal communication skills. All participants believed that further training in suicide risk assessment specific to physiotherapy practice would be beneficial. In addition to training, one participant also reported that they wanted to work more closely with mental health professionals to improve their skills and improve client outcomes.

*It's confronting at times… I used to struggle with it*, *not knowing how to approach it*, *and not knowing what to do…*. *But [after] doing it for over 10 years… I'm now used to it in some ways*. *(IP-4)*

*We [physiotherapists] could be upskilled in the area and probably still save a lot more lives or harm than what we currently do*. *(IP-1)*

*The best thing for the patient… [would be the] opportunity to work together with psychologists and do joint consults … [Joint consults with psychologists] would improve my skills in assessing all those psychological factors and [benefit] the patient*. *(IP-3)*.

Self-care in response to the discomfort experienced by participants when working with clients with suicidal thoughts and behaviours was only briefly mentioned. Participants reported using a range of strategies, including debriefing with their partner, their colleagues, and/or their personal mental health professional to support their mental health and wellbeing.

*My wife is a [health professional]*, *so occasionally [I debrief] with her*. *(IP-4)*

This theme provided an insight into the psychological effects of working with clients with suicidal thoughts and behaviours and to the experience of navigating the middle space. Physiotherapy practice requires physical, intellectual and emotional components, yet the emotional component is rarely discussed [62]. Several participants in the current study believed that their mental health had been affected by the challenges faced by working with clients in the middle space. The discomfort felt by many of the participants entering the middle space and encountering clients with suicidal thoughts and behaviours related to a lack of training in mental health and suicide risk assessment relevant to physiotherapy practice. This finding is consistent with McVey et al. [29], who found that participants lacked confidence in suicide risk assessment likely due to a lack of training. Participants in the current study spoke of feeling underprepared to navigate this space, which is unsurprising given that historically, physiotherapy training has focused heavily on physical approaches, exercise rehabilitation and electrophysical agents with only minor attention to training in psychology [63]. Recent research, and the use of different models of care, such as the biopsychosocial approach in physiotherapy practice, have pushed the physiotherapy profession into new areas such as psychology and mental health [24]. Alexanders [63] theorised that, as a consequence of a lack of training in psychology, physiotherapists might feel underprepared to assess and manage psychological factors. In a systematic review, Holpainen et al. [64] identified that physiotherapists feel underprepared and uncomfortable dealing with psychosocial factors when implementing psychologically informed practice. Andrew et al. [21] found that physiotherapists expressed little confidence in their ability to competently manage people with severe and persistent mental illness; largely due to a lack of training in mental health. Our findings are consistent with these studies.

### Recommendations and implications for physiotherapy practice

Nicholls [65, p. 256] argued that physiotherapists "may find that they need to explore territory occupied by others [such as] … those in the arts, humanities, physical and social sciences" in the future. Recently, there has been a push for physiotherapists to consider the holistic needs of their clients and to integrate psychosocial approaches to practice in line with the biopsychosocial model of care [24, 66]. Theme three: the middle space casts considerable doubt over the preparedness of physiotherapists to work with clients with suicidal thoughts and behaviours and who are at a low to medium risk of suicide, particularly early in their careers. Our recommendations are discussed below.

### Recommendation 1

Undergraduate programs for physiotherapists should ensure that graduates achieve core competencies in suicide risk assessment and prevention.

We believe that a review of the graduate competency standards and program accreditation standards in physiotherapy is warranted. The undergraduate Australian physiotherapy curriculum would benefit from, at a minimum, basic training in suicide risk assessment. As an interim measure, requiring physiotherapists to complete mandatory training as part of their registration may not only give physiotherapists more confidence in situations where clients disclose suicidal thoughts and behaviours, but may also prevent the unnecessary loss of life.

### Recommendation 2

Postgraduate physiotherapy qualifications and professional development courses relevant to psychology and mental health should ensure that physiotherapy graduates achieve advanced competencies in suicide risk assessment and prevention appropriate to their extended scope.

Physiotherapists who are extending their scope of practice to include psychologically informed practice require skills not only for treatment (such as graded exposure), and communication (such as motivational interviewing), but also skills in suicide risk assessment and prevention. Based on this study and the broader literature, the competencies required to practice in the middle space include, at a minimum, being able to complete a suicide risk assessment and a brief psychiatric/mental health assessment.

### Recommendation 3

All suicide prevention training recommended above needs to acknowledge the strength of the physiotherapist-client relationship and be specifically designed for physiotherapists.

Although some of the behaviours of the physiotherapists in this study are not reflective of contemporary mental health practice (for example, the delay in suicide risk assessment), it is important to understand the importance of the physiotherapist-client relationship to suicide prevention. Physiotherapists are uncovering suicidal thoughts and behaviours through small talk, conversational-style interviewing and a trusting practitioner-client relationship. Future suicide prevention training should build on these strengths.

### Limitations and future directions

The recommendations from this study were based on a small sample of physiotherapists who were all from regional NSW and Victoria (Australia) and who were all employed in private practice. Therefore, the recommendations may not reflect the experiences of physiotherapists practicing in public healthcare settings or other regions or countries. The participants also had an average of twelve years of clinical experience; thus, these findings may not reflect the experiences of early-career physiotherapists. A further limitation was that we only recruited physiotherapists with experience of the phenomenon. We believe that further research is required to i) more deeply explore the themes and sub-themes identified in this study; ii) explore if and how the experiences of physiotherapists differ depending on the context of their practice and their level of experience and, iii) investigate the attitudes and practices of physiotherapists who report not having had contact with clients with suicidal thoughts and behaviours.

## Summary and conclusion

The findings offer insights into the experiences of experienced physiotherapists working in private practices in regional NSW and Victoria with clients with suicidal thoughts and behaviours. Physiotherapists believed that trust was essential in facilitating the disclosure of suicidal thoughts and behaviours. However, the primary purpose of developing trust was to create a therapeutic relationship, not to facilitate the disclosure of suicidal thoughts and behaviours. Physiotherapists also reported intentionally adapting their communication style to be more conversational to encourage clients to disclose issues or challenges affecting their physical recovery. Physiotherapists in this study felt relatively competent managing clients with a high suicide risk because they believed there to be one course of action: referral to mental health support to ensure client safety. Similarly, physiotherapists felt confident in managing clients with no identified risk of suicide. Feelings of uncertainty and low confidence emerged during encounters with clients considered to be at low or medium risk of suicide. These feelings of discomfort and uncertainty resulted from physiotherapists lacking the skills required to navigate the middle space that sits in the centre of the broader continuum of clients, ranging from those perceived to be experiencing no foreseeable risk of suicide to those experiencing high risk. The existence of the middle space in physiotherapy practice, as highlighted in this research warrants further attention. The primary recommendation from this study is that all physiotherapists would benefit from suicide prevention training.

## Supporting information

S1 Table Standards for reporting qualitative research. (DOCX)

S1 Text Focus group and interview guide. (DOCX)
