# Supplementary material for: Trust, conversations and the ‘middle space’: A qualitative exploration of the experiences of physiotherapists with clients with suicidal thoughts and behaviours
Source: PLoS One. 2020 Sep 10;15(9):e0238884. doi: 10.1371/journal.pone.0238884 (PMC7482971; doi:10.1371/journal.pone.0238884)
Supplement: S1 Text — (DOCX) [file pone.0238884.s002.docx]

**S1 Text. Focus group and interview guide**

Initial questions used to explore the focus group participants’ experiences.

1. Can you all reflect on a time when you had treated a client with suicidal thoughts and behaviours? I would like each of you to tell me about that in as much detail as possible.
   1. You mentioned __________, can you tell me how that experience made you feel?
2. What made you think that your client had suicidal thoughts?
   1. What suicide risk factors have you observed in your clients?
3. When you have suspected that a client may have had suicidal thought and behaviours what did you do? How did it make you feel?
4. When you have suspected that a client may have suicidal thoughts and behaviours, do you investigate further and if so, what does this look like?
5. Did you directly ask a client if they had suicidal thoughts and behaviours?
   - 1. If so, how did this make you feel?
     2. If not, if you could go back in time would you ask if they were suicidal?
6. Knowing that your client was contemplating suicide, how did that make you feel?
7. Can you tell me what you did to manage any clients who have had suicidal thoughts?
   1. How did managing a client with suicidal thoughts and behaviours make you feel?
   2. Can you tell me about how confident you feel with completing a suicide risk assessment?
8. Are there any barriers or issues that you have experienced when engaging, detecting, assessing, and managing clients with suicidal thoughts and behaviours?
9. How do you feel after seeing a client that was having suicidal thoughts and behaviours
10. With your experience with clients with suicidal thoughts and behaviours, how do you feel about engaging, detecting, assessing, and managing clients with suicidal thoughts and behaviours

Revised questions and prompts used to explore the focus group participants’ experiences.

1. Can you reflect on a time when you had treated a client with suicidal thoughts and behaviours in as much detail as possible?
   1. You mentioned __________, can you tell me how that experience made you feel?
2. What made you think that your client had suicidal thoughts?
   1. What suicide risk factors have you observed in your clients?
3. When you have suspected that a client may have had suicidal thought and behaviours what did you do? How did it make you feel?
4. When you have suspected that a client may have suicidal thoughts and behaviours, do you investigate further and if so, what does this look like?
5. Did you directly ask a client if they had suicidal thoughts and behaviours?
   - 1. If so, how did this make you feel?
     2. If not, if you could go back in time would you ask if they were suicidal?
6. Knowing that your client was contemplating suicide, how did that make you feel?
7. Can you tell me what you did to manage any clients who have had suicidal thoughts?
   1. How did managing a client with suicidal thoughts and behaviours make you feel?
   2. Can you tell me about how confident you feel with completing a suicide risk assessment?
8. Are there any barriers or issues that you have experienced when engaging, detecting, assessing, and managing clients with suicidal thoughts and behaviours?
9. After seeing a client that have perceived as having had perceived as suicidal thoughts, how do you feel after the session?
10. With your experience with clients with suicidal thoughts and behaviours, how do you feel about engaging, detecting, assessing, and managing clients with suicidal thoughts and behaviours
11. Can you tell me about the relationship between yourself and clients who you have identified as having suicidal thoughts and behaviours?
12. Participants from the focus group talked about trust. Does your experience with clients with suicidal thoughts and behaviours involve trust?
    1. Sample quote: *“It’s all about establishing that trust in that relationship, which is probably why people will open up to you about how they're feeling; they may not have disclosed [STBs] to other people.”*
       1. Is the above quote consistent with your experience? Is this consistent or not with your experience? If so, can you explain why? If not, why?
13. Communication was also a theme brought up by participants from the focus group. Could you please tell me about your experience with clients with suicidal thoughts and behaviours in terms of communication?
    1. Sample quote: *While we are doing the manual stuff we’re not sitting there in a quiet room… There is opportunity to pick up on things and to explore that in conversation with somebody. It is through that mechanism of conversation, we tend to find out those sorts of things with people.*
       1. Is the above quote consistent with your experience? Is this consistent or not with your experience? If so, can you explain why? If not, why?
14. Participants from the focus group spoke about their scope of expertise in relation to mental health and suicide. Can you please tell me about your scope of expertise based on your experience with clients with suicidal thoughts and behaviours?
    1. Some participants from the focus group found themselves out of their comfort zone when supporting clients with suicidal thoughts and behaviours. Is this consistent or not with your experience? If so, can you explain why? If not, why?
    2. Sample quote: “*I think that we sometimes do get caught up in becoming counsellors, and we're not equipped to be counsellors or mental health workers. We're good at, I think, screening and being able to see if somebody's at risk and hopefully being out to move them along, but knowing our limitations is so important.”*

*“How do you draw that line?” (Facilitator).*

*“I find that really difficult.”*

- - 1. Is the above quote consistent with your experience? Is this consistent or not with your experience? If so, can you explain why? If not, why?
